# Interactions of Gold and Silver Nanoparticles with Bacterial Biofilms: Molecular Interactions behind Inhibition and Resistance

**DOI:** 10.3390/ijms21207658

**Published:** 2020-10-16

**Authors:** Abhayraj S. Joshi, Priyanka Singh, Ivan Mijakovic

**Affiliations:** 1 The Novo Nordisk Foundation Center for Biosustainability, Technical University of Denmark, 2800 Kgs. Lyngby, Denmark; abshjo@biosustain.dtu.dk (A.S.J.); prisin@biosustain.dtu.dk (P.S.); 2 Department of Biology and Biological Engineering, Division of Systems and Synthetic Biology, Chalmers University of Technology, SE-412 96 Göteborg, Sweden

**Keywords:** biofilm, antimicrobials, gold nanoparticles (AuNPs), silver nanoparticles (AgNPs), biofilm inhibition, molecular interactions

## Abstract

Many bacteria have the capability to form a three-dimensional, strongly adherent network called ‘biofilm’. Biofilms provide adherence, resourcing nutrients and offer protection to bacterial cells. They are involved in pathogenesis, disease progression and resistance to almost all classical antibiotics. The need for new antimicrobial therapies has led to exploring applications of gold and silver nanoparticles against bacterial biofilms. These nanoparticles and their respective ions exert antimicrobial action by damaging the biofilm structure, biofilm components and hampering bacterial metabolism via various mechanisms. While exerting the antimicrobial activity, these nanoparticles approach the biofilm, penetrate it, migrate internally and interact with key components of biofilm such as polysaccharides, proteins, nucleic acids and lipids via electrostatic, hydrophobic, hydrogen-bonding, Van der Waals and ionic interactions. Few bacterial biofilms also show resistance to these nanoparticles through similar interactions. The nature of these interactions and overall antimicrobial effect depend on the physicochemical properties of biofilm and nanoparticles. Hence, study of these interactions and participating molecular players is of prime importance, with which one can modulate properties of nanoparticles to get maximal antibacterial effects against a wide spectrum of bacterial pathogens. This article provides a comprehensive review of research specifically directed to understand the molecular interactions of gold and silver nanoparticles with various bacterial biofilms.

## 1. Introduction

**Figure 1:**
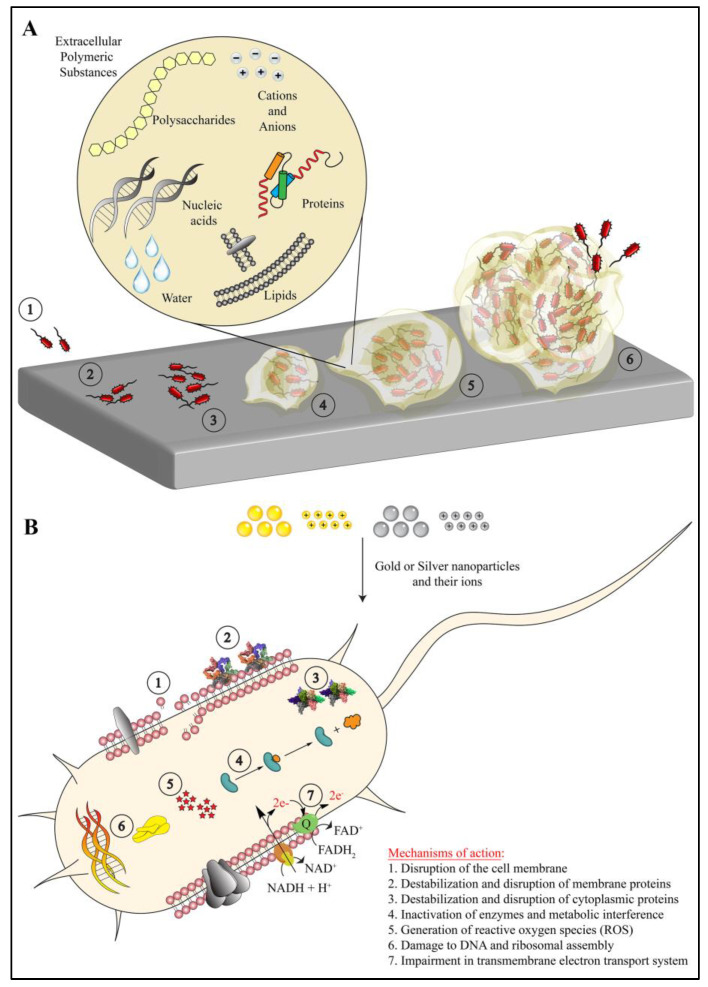
(**A**) The process of biofilm formation in which the bacterial cells approach a biological or non-biological surface (1), adhere firmly (2), multiply (3) and secret extracellular polymeric substances (4). Eventually, this whole mass (i.e., biofilm) grows (5 and 6) into a 3D complex structure that contains nucleic acids, polysaccharides, lipids, proteins, ions and water (magnified circle). (**B**) The mechanism of antibacterial action of gold nanoparticles, silver nanoparticles, and their respective ions, which act on seven specific targets in bacterial cells. The sizes of molecules and bacteria are not to scale, and they are represented arbitrarily for schematic representation only.

Bacteria are one of the prime and essential components of nature’s ecosystem, which perform significant macro-level work at the micron-level [1]. Indigenous to the places from high mountains to deep oceans, cold freezing ice lands to high-temperature volcanic regions, they are arguably the most adaptable living species on Earth [2]. Bacteria exist either in the form of individual cells (planktonic cells) or in the form of biofilms [1,3,4,5,6]. Biofilm is a complex three-dimensional (3D) multilayered structure, formed by multiple planktonic or aggregated bacterial cells by secreting extracellular polymeric substances (EPS) on biological or non-biological surfaces [1,4,5]. Biofilms are formed by single as well as mixed species of bacteria. Depending on the bacterial strains and environmental factors, biofilms show variable physicochemical properties [1,3,4,5]. However, irrespective of bacterial species and environment, all biofilms share some common properties. Biofilms have the tendency to interact specifically (e.g., ligand–receptor interactions) or non-specifically (e.g., ionic and electrostatic interactions) with biological or non-biological surfaces in order to adhere firmly [3,5]. All biofilms consists of EPS (containing several components) that provide a unique 3D structure with a heterogeneous microenvironment suitable for microbial growth and survival [4]. Depending on growth, availability of nutrients and the surrounding environment, all biofilms acquire a peculiar architecture with varying sizes and shapes (mushroom, flat colonies, pillars, filaments, etc.) [1,3]. Biofilms, irrespective of shapes and sizes, show viscoelastic nature that protect bacteria from mechanical stress [1,5]. All biofilms show a growth and maturity cycle that starts with adherence of bacterial cells and ends with release of new bacterial cells in the surrounding medium (Figure 1A) [6]. During formation and maturation, biofilm also serves as a mediator of cell signals as well as a medium for metabolic activities [6].

### 1.1. Bacterial Biofilms and the Components of Biofilms

Biofilms have diverse functions owing to its multicomponent structure. These components can be categorized into six categories: polysaccharides, proteins, nucleic acids, lipids, water and ions (e.g., cations like Ca^2+^ or Mg^2+^ and anions like Cl^−^ or PO_4_^2−^) [1,3,4,5]. The composition of biofilm depends on species of bacteria, surrounding environment and availability of nutrients. Biofilm composition also changes depending on the number of bacterial species embedded within mixed biofilm [1]. With the help of extracellular polysaccharides like α-mannans, β-glucans, fructans, xanthan, poly-γ-glutamate, alginate, Psl, Pel and some proteins like the biofilm-associated protein (BAP), lectins, fibronectin binding proteins (FnBPs) along with interacting ions like Ca^2+^ or Mg^2+^, biofilm helps bacterial cells to adhere strongly on biological or non-biological surfaces [1,3,4,5]. Polymers like alginate, Psl and Pel also help in maturation as well as maintaining architecture and viscoelasticity of the biofilm together with ions and water [3,4,5]. Via amyloid-forming proteins and polysaccharides, biofilms maintain cohesion within molecules of EPS, and this eventually helps in formation and maturation of the biofilm. Structural proteins and proteinaceous outgrowths (e.g., fimbriae) contribute to adhesion along with the aforementioned polysaccharides [1,3,4,5]. The polysaccharides and proteins of biofilm restrict movement of the newly dividing bacterial cells within biofilm and keep them in close vicinity to each other [1,5]. With this restriction and extracellular DNA (eDNA), biofilms mediate and regulate cell-to-cell communication, also known as quorum sensing (QS) [1,3,4,5]. eDNA present in the biofilm offers protection by means of its inherent antimicrobial activity as well as through horizontal gene exchange, which is essential in developing resistance against foreign bactericidal molecules (e.g., classical antibiotics) [1,3,4,5]. Biofilm holds many enzymes, which catalyze lytic and redox reactions that are essential for making nutrients available, clearing dead cell debris and nullifying toxic molecules present in the biofilm microenvironment. Biofilms also cause biofouling or biocorrosion with the help of these enzymes [1,3,4,5]. Biofilm absorbs carbon-, nitrogen- and phosphorous-containing compounds as well as inorganic ions and oxygen from surrounding environment. These compounds and/or their constituents serve as an energy source and as a primary reactant for metabolism for embedded bacterial cells [1,3,4,5]. With the help of lipids like surfactin and serrawettin, biofilm acquires hydrophobic properties that are essential for adhesion, absorption of hydrophobic nutrients and maintenance of surface tension at the interfaces [3]. Owing to the absorption by extracellular polysaccharides and proteins, biofilms retain water and form a slimy mass, which provides protection to bacterial cells from mechanical stress as well as from desiccation [1,3,4,5]. Biofilms also serve as the center for recycling metabolic waste. Biofilms are involved in maintaining and releasing dormant bacterial cells, which eventually leads to improved pathogenicity and infection or disease progression [1,3,4,5]. Additionally, biofilms offer resistance against many antibacterial entities by (i) inhibiting their diffusion by molecular sieve action of interconnected EPS, (ii) enzymatic degradation, (iii) increasing the hydrophobic character (e.g., secretion of BslA protein in *Bacillus* species) and (iv) genetic modification (horizontal gene transfer) [1,4,5]. In short, these components work in concert to provide a mechanically stable and functionally dynamic microenvironment of biofilm. Hence, biofilms are a widespread mode of survival for bacteria, enabling them to thrive and spread in the surrounding environment. Since the bacterial biofilms and their constituents are crucial for bacterial survival in contact with humans, it represents a logical target for new antibacterial treatments.

### 1.2. Current Status of Biofilm Inhibition

A recent study suggested that a total of 407 antibiotic projects from small-scale and medium-scale enterprises are in preclinical testing phases across the globe. These projects have been directed towards finding small molecules, potentiators for classical antibiotics, antibodies, vaccines, immunomodulators, selective bacteriophages against pathogens, modulators for normal microbial flora and nanobiotechnological antibacterial agents [7]. Comprehensive analysis of the multicomponent nature of the biofilm has chalked out few excellent targets for combating microbial infections and infectious diseases [1,8,9]. The biofilm inhibition approaches can be categorized into four classes: (i) targeting bacterial adhesion and EPS components, (ii) targeting biofilm metabolism, (iii) facilitating biofilm dispersal and (iv) targeting dormant cells [7,8,9,10]. The early stages of biofilm formation (i.e., bacterial adhesion and EPS synthesis) can be targeted using molecules such as bacterial adhesion inhibitors (mannoside derivatives), cyclic-di-GMP pathway targeting molecules (e.g., cephalosporin-3′-diazeniumdiolate) and inhibitors of lipopolysaccharide (LPS) synthesis enzymes (e.g., LpxC inhibitors) [7,8,9,10]. Early biofilms and mature biofilms can be eradicated using structural protein inhibitors (e.g., ring-fused 2-pyridones), eDNA-targeting molecules (e.g., alginate derivatives), QS inhibitor peptides (e.g., AIP-I or RIP), EPS-degrading enzymes (e.g., glucanohydrolases or dispersin) and monoclonal antibodies (e.g., Ebp A) [7,8,9,10]. The metabolism of the biofilms can be disrupted using exogenously administered amino acids (e.g., L-arginine or L-methionine) or metals (e.g., gallium), which hamper particular metabolic pathways, promote biofilm disassembly and make them vulnerable to classical antibiotics [7,8,9,10]. Apart from this, attempts have been made to formulate vaccines against common pathogens such as *Staphylococcus aureus*, which was effective against planktonic bacteria as well as a mature biofilm [8]. Moreover, various small organic molecules such as chlorhexidine and novel antimicrobial peptides (AMPs), metallic nanoparticles (e.g., nanoparticles made up of gold, silver, copper, cerium), graphene nanosheets and quantum dots (with or without other antibacterial molecules) have shown great potential in preclinical and clinical phases against various Gram-positive and Gram-negative bacterial biofilms [7,8,9,10,11,12]. Further, in the case of surgical implants, surface modification (e.g., silver coating of catheter) has reduced the chances of biofilm formation significantly. This strategy has resulted in the possibility of increasing the life of implants and other biomaterials [8]. In addition, mechanical means such as water jets/sprays, ultrasound waves and photothermal irradiation have been proven effective against biofilms in certain cases [8,9]. Overall, at present, several therapeutic modes are available for biofilm inhibition. Due to advances in microbiology and biofilm understanding, it is possible to devise multifaceted approaches by selecting particular antimicrobial treatment modes from the aforementioned list to maximize synergy.

## 2. Gold and Silver Nanoparticles as Antimicrobial Agents

Over the last decade, the resistance to almost all classical antibiotics and the lack of availability of novel antimicrobial molecules has directed the efforts of researchers towards exploring nanotechnological measures against microbial infections for therapeutic applications [7,8,9,10,11,12,13,14,15]. To date, several types of nanoparticles have shown their effectiveness in killing bacteria and eradicating biofilms more efficiently than classical antibiotics. In general, antimicrobial nanoparticles can be categorized as (i) metallic nanoparticles (e.g., gold and silver nanoparticles), (ii) polymeric nanoparticles (e.g., chitosan nanoparticles), (iii) carbon-based nanoparticles (e.g., graphene nanosheets), (iv) lipidic nanoparticles (e.g., liposomes), (v) non-metallic inorganic nanoparticles (e.g., silica nanoparticles) and (vi) protein nanoparticles (e.g., albumin nanoparticles) [14,15]. In this article, we have focused on gold nanoparticles (AuNPs) and silver nanoparticles (AgNPs) and their molecular interactions with bacterial biofilms. In nanoparticle form, both gold and silver offer several advantages. Briefly, a high surface area to volume ratio, amenability to surface modification, small size (less than 10 nm), inert nature, biocompatibility and biosafety with easy clearance from tissue make these nanoparticles a suitable choice for antimicrobial therapy and drug delivery [13,14,15]. Other metallic nanoparticles made of copper, zinc, titanium, cerium and their respective oxides offer similar advantages [16]; however, their toxic effects in humans and the environment outweigh their advantages [17,18]. In addition, the literature showing antimicrobial effects of other metallic nanoparticles and possible mechanistic details behind their action is very limited. The extent of research involving AuNPs and AgNPs in this area is extensive. Additionally, current advances in nanotechnology, chemistry and biotechnology allow the synthesis of AuNPs and AgNPs by simple, cheaper and green methods along with their fine-tuning and surface modification for improved and synergistic action. This, in particular, holds tremendous potential in developing a robust and highly specific antimicrobial treatment modality [19].

While exerting their antimicrobial action, AuNPs and AgNPs undergo three important steps when they are in close vicinity to the biofilm or co-incubated with the biofilm [13]. First, these nanoparticles interact with the biofilm surface after approaching it from bulk phase. The nanoparticles, depending on their surface chemistry, charge and hydrophobicity, interact with lipids, LPS or proteins of the bacterial cell membrane [13]. Subsequently, depending on this interaction, the nanoparticles gain entry into the biofilm. The penetration of nanoparticles depends on many factors such as biofilm maturity, biofilm surface composition and chemistry, nanoparticle size, surface charge, surface chemistry and nanoparticle concentration [13]. After penetration, the nanoparticles as a whole or as ions (gold ions (Au^+^) and silver ions (Ag^+^) leached from nanoparticles) migrate internally in order to interact with biofilm components and cellular components [13]. Hence, the antimicrobial action of AuNPs and AgNPs relies on disrupting several biofilm and bacterial components [13,14,15]. One can modify the nanoparticles to achieve a preferential mode of action against single or multiple bacterial species. The interactions involved in approaching the biofilm surface, penetration and internal migration will be discussed in subsequent headings in this article. In general, the electrostatic, hydrophobic, hydrogen-bonding and Van der Waals attraction interactions are involved in all these processes (Table 1) [20].

AuNPs and AgNPs exert their antimicrobial action in several ways (Figure 1B). The mode of their bacteriostatic/bactericidal action may or may not be specific against a molecular target, but they largely show summation of several molecular events that tend to inhibit or modify or disrupt various targets in the planktonic bacteria and biofilms. As shown in Figure 1B, AuNPs and AgNPs eradicate bacterial biofilms via seven distinct modes of action [11,12,14]. AuNPs and AgNPs interact with the lipid bilayer as well as the LPS of bacteria, and they disrupt the bacterial membrane via membrane fluidization. Additionally, they modify the hydrophobicity of the membrane. Upon disruption, the nanoparticles and ions leached from them gain access to the cytosol through the membrane pores. The cytoplasmic content also leaks out through these pores. Further, these nanoparticles also destabilize the membrane proteins (e.g., efflux pumps), cytoplasmic structural proteins (e.g., actin) and various enzymes (e.g., oxidoreductase family), resulting in inhibiting their functions and leading to overall metabolic impairment. This leads to structural collapse, poor adhesion to the surface, inhibition of metabolic pathways and eventually bacterial cell death. Further, the ions leached from AuNPs and AgNPs generate reactive oxygen species (ROS), which create oxidative stress. The bacterial cell fails to cope and eliminate the excess oxidative stress created by superoxide radicals, hydroxyl radicals and hydrogen peroxide. These radicals also change the permeability of the bacterial membrane. Additionally, the ions leached from these nanoparticles also interact with free amino, carboxyl and mercapto groups of proteins and nucleic acids. AuNPs and AgNPs also inhibit the electron transport chain of bacterial cells, which leads to impaired ion exchange across the membrane, membrane destabilization and reduced metabolic activity. Altogether, these effects lead to bacterial cell death and biofilm elimination [11,12,14].

## 3. Interaction of Gold and Silver Nanoparticles with Biofilm Components

### 3.1. Interactions with Biofilm Nucleic Acids

eDNA plays a vital role in bacterial adhesion, bacterial aggregation within biofilm, biofilm formation, biofilm structure, biofilm integrity as well as intercellular communication or QS for transfer of genetic information [1,3,5]. Therefore, eDNA has proven to be an excellent target for eradicating bacterial biofilms [1]. AuNPs and AgNPs have affinity towards bacterial DNA, and they show different interactions with eDNA depending on the microenvironment of the biofilm. It is well-known that when a nanoparticle enters into a biological media, almost immediately it acquires a corona over its surface comprising several molecules of biological origin in either monolayer or multilayer form [21]. Thus, the interaction of AuNPs and AgNPs with any component of biofilm is also affected by this corona [21]. Apart from this, it has been shown that the change in salt or varying concentrations of a salt leads to changes in the kinetics of interaction of DNA with AuNPs and AgNPs [22]. Thus, determining exact interactions of biofilm components with pure gold or silver surfaces is practically not feasible at present. Nonetheless, in vitro experiments done with bare AuNPs and AgNPs (nanoparticles without surface modifications and/or without corona on the surface) showed that, in presence of salts, DNA interacts with nanoparticles via Van der Waals forces as well as hydrophobic forces and gets adsorbed, preventing aggregation of nanoparticles in solution [22,23]. With extrapolation of this scenario in the context of biofilm inhibition, we can safely assume that various ions present in the biofilm matrix may control the interaction and adsorption of eDNA on bare AuNPs and AgNPs. However, the chances of bare nanoparticles being present in the biofilm are very limited, which makes this type of interaction a rare phenomenon.

**Figure 2:**
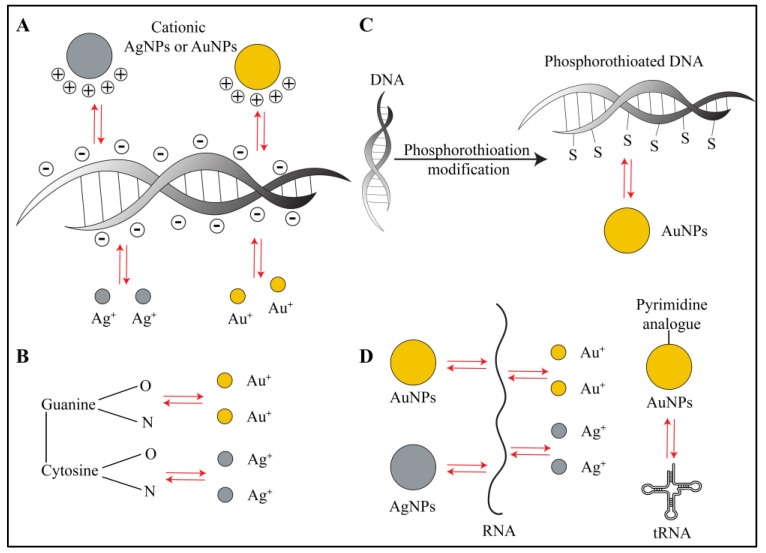
Schematic representation of interactions between biofilm nucleic acids and gold or silver nanoparticles. (**A**) Cationic gold nanoparticles, silver nanoparticles and their respective metal ions show electrostatic interactions, hydrophobic interactions and Van der Waals interactions with polyanionic eDNA in the biofilm. (**B**) The metal ions (Au^+^ and Ag^+^) in particular show affinity towards G-C base pairs of eDNA, where they preferentially interact with oxygen and nitrogen atoms via short-range Van der Waals forces and hydrophobic forces. (**C**) In order to withstand oxidative damage, bacteria undergo phosphorothioation modification of DNA. Gold nanoparticles show highly specific gold–sulphur (Au-S) bonding interactions with such eDNA. (**D**) Both gold and silver nanoparticles and their ions show electrostatic interactions and Van der Waals interactions with bacterial RNA and tRNA. Red arrows in the figure indicate different types of interactions.

As eDNA possesses a polyanionic nature, in the case of AuNPs and AgNPs that are coated with positively charged molecules, electrostatic interactions play the primary role. Carnerero et al. showed that AuNPs exhibit covalent as well as non-covalent interactions with a polyanionic eDNA backbone [24]. Additionally, Carnerero et al., Koo et al. and Jiang et al. proved that the gold and silver ions leached from respective nanoparticles also interact with oxygen and nitrogen atoms of DNA bases via short-range hydrophobic forces and Van der Waals forces [24,25,26]. However, electrostatic interactions dominate over Van der Waals and hydrophobic interactions. In a recent interesting study, which was done with femtosecond spectroscopy, gold ions showed inhibition of biofilm formation in Gram-negative bacteria [27]. The authors stated that the interaction and subsequent binding of cellular DNA to gold ions was a primary reason behind DNA damage instead of ROS generated by AuNPs. ROS-mediated oxidative damage induces DNA repair mechanisms in bacteria. However, mutant bacterial strains deficient in DNA repair mechanisms showed vulnerability similar to that of wild-type strains to gold ions. Based on this observation, the authors attributed biofilm inhibition to the interactions of gold ions with cellular DNA and its subsequent damage [27]. Similar observations have been made in the case of silver ions, where in vitro experiments showed cooperative binding with DNA [26] (Figure 2A,B). Interestingly, it has been proven that phosphorothioation (PT) modification of DNA in bacteria makes them stable against oxidative damage in unfavorable environments [28]. In such cases, treating biofilms that contain phosphorothioated DNA with AuNPs would result in a strong adsorption owing to favorable Au-S chemistry [24,25]. It is generally believed that specific and strong Au-S bonding interactions would play a predominant role in the inhibition of eDNA function along with electrostatic and hydrophobic interactions (Figure 2C).

AuNPs and AgNPs also interact with cellular RNA as well as extracellular RNA in biofilms [29,30] (Figure 2D). Computational studies have shown that the gold and silver ions leached from respective nanoparticles have affinity towards RNA bases and base pairs [31]. Proteomics and transcriptomic studies performed on AuNPs and Gram-negative bacterial biofilms reveal that the nanoparticles directly interact and bind with tRNA inside the bacterial cell due to a pyrimidine analogue conjugated on the AuNP surface [30]. This binding causes inhibition of the ribosomal functions, death of bacterial cells and eventually leads to significant reduction in biofilm [30]. In another study, Tian et al. showed that AgNPs directly interacted with small regulatory RNA and changed the RNA expression profile in *S. aureus*, leading to decreased biofilm formation and fibronectin binding [29]. In general, AuNPs, AgNPs and their respective ions interact with extracellular and cellular DNA and RNA in biofilms, via multiple types of interactions, and achieve antimicrobial activity.

### 3.2. Interactions of Nanoparticles with Biofilm Proteins

**Figure 3:**
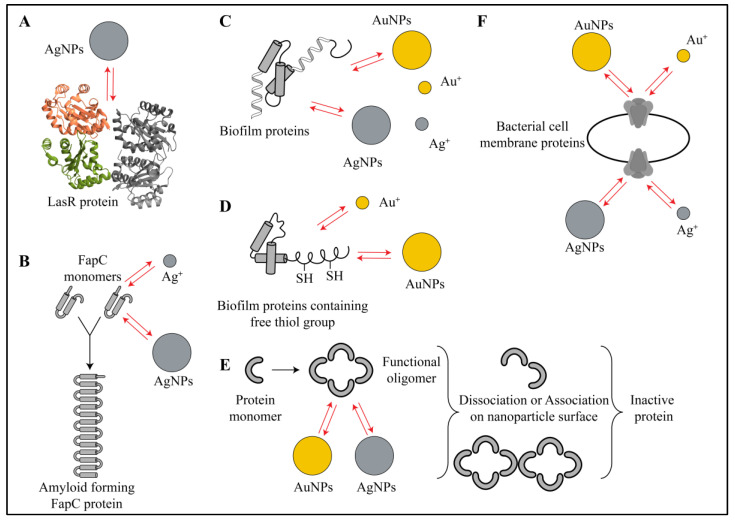
Schematic representation of various interactions between gold or silver nanoparticles and biofilm proteins. (**A**) The metallic nanoparticles (e.g., silver nanoparticles) interact with few proteins that are involved in quorum sensing (e.g., LasR) via electrostatic, hydrophobic and hydrogen-bonding interactions. In this case, nanoparticles and their ions preferentially interact with amino acids at ligand binding sites, making them inactive for cell signaling. (**B**) Gold and silver nanoparticles also interact via electrostatic, hydrophobic and hydrogen-bonding interactions with amyloid-forming proteins such as FapC, sequester its monomers and create defects in bacterial cell structure. (**C**) Several metabolic proteins and enzymes in the biofilm interact with nanoparticles via electrostatic, hydrophobic, hydrogen-bonding, Van der Waals and π–π interactions, causing collapse in the protein structure and eventually making them inactive for metabolism. (**D**) Gold nanoparticles and gold ions show strong Au-S bonding interactions while interacting with thiol-containing proteins of the biofilms. (**E**) Gold and silver nanoparticles also interact with functional oligomers of certain proteins via electrostatic and hydrophobic interactions and lead to their association or dissociation, which ultimately leads to impairment in their function in the biofilm. (**F**) Membrane proteins (e.g., electron transport system) also have similar interactions with metal ions and metallic nanoparticles that leads to loss in integrity and changes in membrane protein functions. Red arrows in the figure indicate all types of interactions.

Extracellular proteins are another essential component of the biofilm matrix. Several structural proteins, membrane proteins and enzymes work in coordination with each other and with other biofilm components to maintain adhesion, cohesion, EPS production, EPS modification, QS and metabolic homeostasis [1,3,5]. Since proteins are involved in very diverse functions, their inhibition would result in a total destruction of biofilm and planktonic bacterial cells. Hence, proteins are one of the attractive targets for finding new antimicrobial therapies. To date, it has been shown that AuNPs and AgNPs, along with their respective ions, interact with several structural proteins, membrane proteins and several enzymes [32,33,34,35,36,37,38,39,40,41,42,43,44,45,46]. In an experimental and molecular docking study, Shah et al. evidenced the diverse action of AgNPs on various proteins of *Pseudomonas aeruginosa* [32]. First, they showed a reduction in the amount of elastase, a QS-mediated virulence factor, caused even by exposure to sublethal doses of AgNPs. Further, they also showed binding and inhibition of three other QS regulator proteins: LasR, Lasl and MvfR. The authors used eugenol-stabilized AgNPs that were synthesized by a green method from a plant extract. The molecular docking studies showed that the amino acids at the active site of LasR exhibited electrostatic (for aspartate) and hydrophobic interactions (for tyrosine, tryptophan and alanine) with the nanoparticles (Figure 3A). The binding efficiency was higher for eugenol-stabilized AgNPs as compared to only eugenol and only AgNPs. For Lasl protein, the docking showed electrostatic interactions, hydrophobic interactions and hydrogen-bonding interactions with the nanoparticles. For MvfR protein, the docking showed only hydrophobic interactions and hydrogen-bonding interactions at the active site. Their overall experimental results gave proof of QS inhibition and biofilm destruction [32]. In a similar study, with homology modeling, Vyshnava et al. showed interactions of four proteins involved in the signal transduction system of *P. aeruginosa* (LasR, QscR, RhlR and Vfr) with AgNPs, which resulted in their binding and inhibition [33]. In this study, it is reported that several amino acids—leucine, aspartate, arginine, lysine, tyrosine, arginine, alanine and tryptophan—from active sites of the four proteins interact with AgNPs [33]. Structural proteins, such as bacterial amyloid FapC in *P. aeruginosa*, form a branched structure that helps in cell–cell adhesion, encasing dormant cells, QS, protection and biofilm integrity [34]. In a study done by Huma et al., AgNPs prevented fibrilization of FapC proteins by interacting with and sequestering FapC monomers (Figure 3B). To support these experimental findings, when the authors performed an in silico analysis, they found that AgNPs interacted via electrostatic, hydrophobic and hydrogen-bonding interactions with several amino acid residues of the FapC protein [34]. In vitro thermodynamic study on branched polyethylenimine coated silver nanoparticles (bPEI-AgNPs) also supported the existence of electrostatic, hydrophobic and hydrogen-bonding interactions as well as changes in the secondary structure of alpha lactalbumin protein due to these interactions [35]. The quantitative proteomic study done by Zhang et al. suggested involvement of AgNPs and silver ions in inhibition of several proteins from the oxidative phosphorylation pathway, nitrogen metabolism, chemotaxis, flagellar assembly, ribosomes and electron transport chain [36] (Figure 3C). Similar observations have been reported for inhibition of efflux pump proteins and QS by AgNPs [37]. Even though these reports did not give concrete experimental proof for the existence of nanoparticle–protein interactions [36,37], there are many publications that have revealed the existence of these interactions [39]. Abdelhamid and Wu gathered and reviewed proteomic data collected by UV–visible spectrometry, Raman spectroscopy, fluorimetry, mass spectrometry, nuclear magnetic resonance (NMR), dynamic light scattering (DLS), circular dichroism, X-ray crystallography and isothermal calorimetry [39]. Their review suggests that the electrostatic, hydrophobic, hydrogen-bonding, Van der Waals and π–π interactions between proteins of Gram-positive as well as Gram-negative biofilms and AuNPs/AgNPs are responsible for the antibacterial action of nanoparticles [39] (Figure 3C). The activity of several envelope proteins and efflux pumps from *Escherichia coli* [40], many enzymes and ATP binding proteins from *S. aureus* [40] and several membrane proteins involved electron transport system of *Bacillus thuringiensis* were inhibited by AgNPs [41]. In the case of AuNPs, proteomic data revealed that nanoparticles were responsible for interacting and inhibiting the thiol-containing proteins via Au-S bonding interactions (Figure 3D). In this study, AuNPs also changed the ratio of oxidized and reduced proteins causing metabolic imbalance in *Mytilus edulis* [42]. Further in *Escherichia coli*, AuNPs inhibited ATPase enzymes and ribosomal proteins leading to bacterial cell death [43]. In a separate report, Chakraborty and Biswas showed the interaction of AuNPs and AgNPs with virulence causing antigenic heat shock protein-18 (HSP-18) of *Mycobacterium leprae* [38]. The authors showed that the AuNPs and AgNPs induced structural changes after interacting with HSP-18, which finally led to changes in its activity. They observed oligomeric association of in the presence of AuNPs, whereas in the presence of AgNPs, the oligomers dissociated, leading to changes in its chaperon-like function (Figure 3E). This change in activity was dependent on interactions shown by nanoparticles with HSP-18 [38]. Only AgNPs were found to be effective in eradicating the biofilm. Authors attributed these results to electrostatic and hydrophobic interactions [38]. In an interesting report, Jena et al. used bimetallic hybrid gold–silver nanoparticles (Au-Ag-NPs) to study their interactions with bacterial proteins [44]. According to this report, the electrostatic interactions between positively charged bimetallic nanoparticles and negatively charged bacterial surfaces promoted adhesion of nanoparticles on the bacterial cell surface. Further, these nanoparticles hindered the functions of membrane proteins involved in the electron transport system of *E. coli* and *Bacillus subtilis* [44] (Figure 3F). In addition, these nanoparticles caused delocalization of MreB protein (a protein from the actin family), leading to increased membrane fluidity and loss of structural integrity. All these effects eventually led to bacterial cell death [44]. To summarize, we can say that several proteins interact through their free functional groups and/or side chains of amino acids with AuNPs and AgNPs. Overall, a combination of electrostatic interactions, Van der Waals interactions, hydrophobic interactions, hydrogen-bonding interactions and π–π interactions between these nanoparticles and bacterial proteins leads to modification of protein structure/activity and ultimately to biofilm inhibition.

### 3.3. Interactions of Nanoparticles with Biofilm Polysaccharides

Polysaccharides present in the biofilm can be categorized in two categories: (a) polysaccharides present in cell walls of bacteria and (b) polysaccharides present in the biofilm structure, which are extracellular and secreted by the bacterial cells while forming the biofilm [1,3,5,47]. Polysaccharides present in the bacterial cell wall offer mechanical strength, structural stability, osmoregulation, virulence property and bacterial cell integrity [1,3,5,47]. On the other hand, extracellular polysaccharides secreted by biofilm-forming bacteria are involved in adhesion, cohesion, formation of protective barrier, maintaining biofilm integrity and maintaining nutrient sources within the biofilm microenvironment while keeping bacterial cells in close proximity for cell–cell interactions [1,3,5,47]. Hence, polysaccharides serve as another excellent target for developing biofilm inhibition strategies. AuNPs and AgNPs interact with cell wall polysaccharides as well as extracellular polysaccharides secreted in the biofilm.

**Figure 4:**
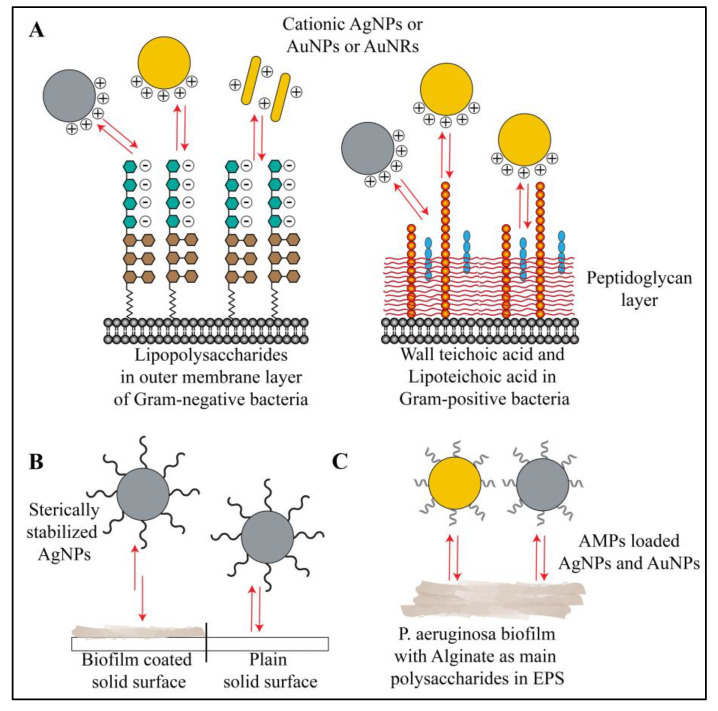
Schematic representation of interactions between gold or silver nanoparticles and polysaccharides of biofilm. (**A**) Bacterial polysaccharides (LPS, teichoic acid and lipoteichoic acid) show electrostatic interactions, hydrogen-bonding interactions and hydrophobic interactions with cationic gold and silver nanoparticles. Neutral and anionic gold and silver nanoparticles show no or minimal interactions under the same experimental conditions. (**B**) In some cases, the nanoparticles stabilized with large, non-ionic polymers (e.g., PVP coated AgNPs) show specific electrosteric repulsive interactions with biofilm polysaccharides. (**C**) Antimicrobial peptide coated gold and silver nanoparticles show electrostatic as well as hydrophobic interactions with biofilm polysaccharides (e.g., alginate in *P. aeruginosa* biofilm). Red arrows in the figure indicate all types of interactions.

The interaction of polyelectrolyte (e.g., anionic polyacrylic acid (PAA) and cationic poly(allylaminehydrochloride) (PAH)) coated gold nanorods (AuNRs) with LPS of various Gram-negative bacteria was tested by refractometric sensing [48]. In this study, authors found that LPS from Gram-negative bacteria like *P. aeruginosa*, *Salmonella enterica* and *E. coli* bind AuNRs, and the binding strength (in terms of number of LPS molecules per nanorod) depends on the interaction of LPS with polyelectrolytes such as PAA/PAH [48]. The authors observed a strong interaction between anionic LPS and cationic NRs (Figure 4A). Authors have suggested that electrostatic interactions played a primary role in LPS interaction with NRs, along with other contributing factors such as LPS composition (percentage of sugars and fatty acids in LPS molecules and the overall negative charge arising from these components), LPS structure and LPS concentration in the cell wall of different bacterial strains. However, in the same report, authors have not reported any interaction between bare AuNRs or gold ions and the LPS of bacterial strains [48]. In another study, Pajerski et al. used Derjaguin–Landau–Verwey–Overbeek (DLVO) theory to explain the attachment efficiency and the interactions of AuNPs with LPS of Gram-negative and Gram-positive bacteria [49]. Using DLS and measuring the zeta potential, the authors concluded that bacteria and nanoparticles interact via electrostatic forces. In their study, the Gram-negative bacteria showed fewer interactions with AuNPs compared to Gram-positive bacteria. This finding was in accordance with the measured zeta potentials of Gram-positive and Gram-negative bacteria (~41 mV versus ~26 mV, respectively). According to DLVO theory, the electrostatic repulsive forces and Van der Waals attractive forces are involved in the interaction of two colloids (nanoparticles and bacterial cells) in an aqueous suspension. In the case of Gram-positive bacteria, the attractive forces dominate the repulsive forces; hence, the AuNPs have a higher adsorption efficiency when binding to lipoteichoic and teichoic acid of the bacterial cell wall. On the other hand, in the case of Gram-negative bacteria, the repulsive forces dominate, diminishing interactions. To some extent, the hydrophobicity of the bacterial cell wall also plays a role in the interaction, but electrostatic forces dominate [49] (Figure 4A). Jeckobson et al. provided evidence for the impact of LPS density and structure on overall interactions of bacterial cells with metallic nanoparticles [50]. Depletion of LPS from the bacterial cell wall by EDTA treatment reduced their interaction and association with cationic AuNPs. By contrast, anionic AuNPs exhibited no or minimal association with Gram-negative bacteria, irrespective of LPS density or structure [50]. Further, the authors explored the role of *O*-polysaccharides. Interaction of LPS with AuNPs is directly proportional to the *O*-polysaccharide content in LPS. This effect is most probably due to many potential anionic sites (more phosphate and carboxylate groups) present in *O*-polysaccharides, which participate in interactions with AuNPs. Overall, the authors showed that the charges on the bacterial cell wall due to the presence of LPS and charges on the AuNPs surface control the electrostatic interactions (attraction or repulsion) between them [50]. In the same line of thought, Caudill et al. have shown that the composition of teichoic acid can alter the interactions of cationic branched polyethylenimine coated gold nanoparticles (bPEI-AuNPs) with LPS of Gram-positive *B. subtilis* [51]. Using ^31^P and ^13^C-NMR, authors showed that bPEI-AuNPs interact electrostatically as well as form hydrogen bonds with oxygen atoms of phosphate groups of teichoic acid residues in LPS [51]. Changes in teichoic acid composition (% of glucose or alanine moieties) lead to alteration in interactions with bPEI-AuNPs. Fewer negatively charged residues in teichoic acid led to reduced interactions with cationic AuNPs [51] (Figure 4A). Similar interactions with LPS have been reported for AgNPs [52,53,54]. In a different study, authors used a positively charged chitosan polymer for coating and stabilizing AgNPs (chitosan-AgNPs). The chitosan layer on AgNPs interacted strongly with the negatively charged LPS molecules [55]. The authors used these interactions in order to make an ultrasensitive sensor for Gram-negative model microorganism *E. coli* [55]. Mitzel and Tufenkji reported an unusual observation where they used quartz sand surface coated with *P. aeruginosa* biofilm for checking biofilm interaction with poly(vinyl pyrrolidone)-coated silver nanoparticles (PVP-AgNPs) [56]. They found that, owing to the non-ionic nature as well as bigger structure, the amount of PVP-AgNPs attached to biofilms was inferior to the amount attached to the clean surface. Authors described this as electrosteric repulsion interactions with large biofilm polymers, such as polysaccharides [56] (Figure 4B). Badawy et al. compared the electrostatic interactions of citrate-coated AgNPs and bPEI-AgNPs and observed that the cationic bPEI-AgNPs had stronger electrostatic attraction, leading to higher bacterial cell toxicity [54] (Figure 4A). This finding emphasizes the importance of surface chemistry of both AuNPs and AgNPs for their fate in the biofilm microenvironment. In the case of exopolysaccharides, concrete reports showing their interaction with AuNPs and AgNPs are not available. However, according to a report published by Dunsing et al., biofilm of the plant pathogen *Pantoea stewartia*, composed purely of polysaccharides, interacted with nanoparticles and hindered their penetration into the biofilm matrix [57]. In a different report by Kalishwaralal et al., the AgNPs reduced synthesis of exopolysaccharides in *P. aeruginosa* and *Staphylococcus epidermidis* biofilms by unknown mechanisms [58]. Antimicrobial peptides (AMPs) are one of the new molecules that have shown tremendous potential against multiple drug resistant bacteria [59]. AuNPs and AgNPs conjugated with AMPs have shown electrostatic and hydrophobic interactions with biofilms, leading to antimicrobial effects [59,60,61,62,63]. The action of AMPs on the surface of AuNPs and AgNPs involve interactions with LPS or extracellular polysaccharides, such as alginate in *P. aeruginosa* [59,64] (Figure 4C). Interestingly, extracellular polysaccharides may also hinder the antimicrobial action of AMP-coated nanoparticles [64]. Overall, polysaccharides from the biofilm matrix and LPS of bacteria preferentially show electrostatic interactions with metallic nanoparticles, owing to their negatively charged nature.

### 3.4. Interactions of Nanoparticles with Biofilm Lipids

**Figure 5:**
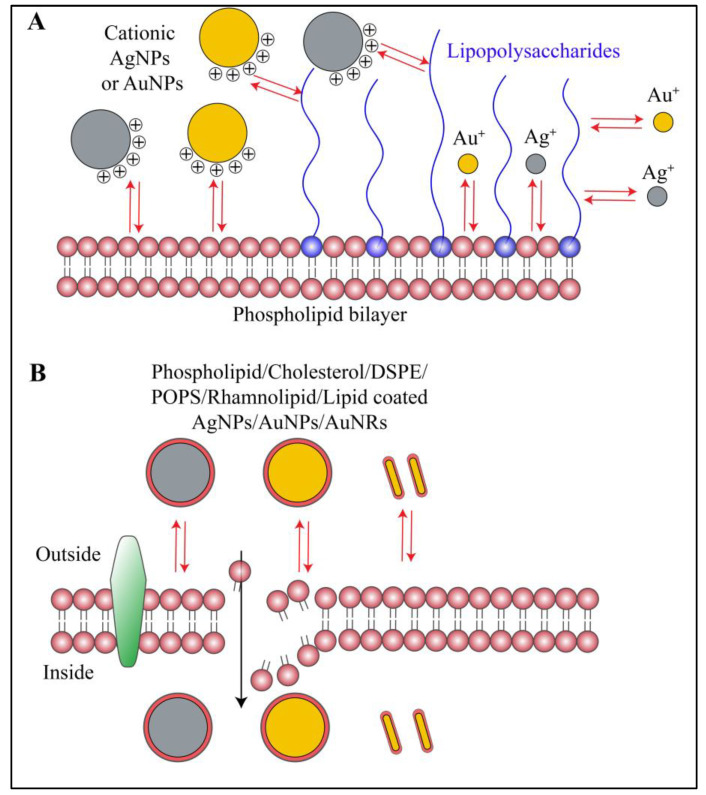
Schematic representation of interactions between biofilm lipids and gold or silver nanoparticles. (**A**) Cationic gold nanorods, gold nanoparticles and silver nanoparticles along with their respective ions interact with biofilm lipid and lipopolysaccharides via electrostatic and hydrophobic interactions. Whereas, anionic or neutral nanoparticles or nanorods show minimal or no interactions at all. (**B**) Gold nanorods, gold nanoparticles and silver nanoparticles coated with lipid moiety or any other hydrophobic moiety preferentially show hydrophobic interactions with biofilm lipids. Red arrows in the figure indicate different types of interactions.

Apart from hydrophilic components like polysaccharides, nucleic acids and proteins, the hydrophobic properties of biofilm comes from lipids, lipopolysaccharides and surfactants secreted by bacteria [1,3,5]. Lipids generated from lysed dormant bacterial cells, lipids of dormant live bacterial cells and the lipidic components of lipopolysaccharides constitute this class of biofilm components. Although the biofilm lipids are relatively less described in the literature, they are essentially involved in adhesion, cohesion, supply of nutrients and transport of metabolites [1,3,5]. Due to their hydrophobic nature, lipids are engaged in different types of interactions with AuNPs and AgNPs. For example, in a report published by Bakri et al., the authors proposed interactions of lipid-coated AuNPs with the biomembrane of bacteria [65]. They used 1,2-distearoyl-sn-glycero-3-phosphorylethanolamine-coated gold nanorods (DSPE-AuNRs) against *P. aeruginosa* and suggested that DSPE interacts with bacterial lipid membrane via hydrophobic interactions and destabilizes it (Figure 5A,B). These hydrophobic interactions, the increment in fluidity of membrane (also known as lipid-mixing effect) and membrane destabilization altogether lead to internalization of AuNRs, which further hampers other metabolic processes and kills the bacterial cells [65]. Similar observations have been reported by the same research group in a different report, where they used phospholipid-PEG and cholesterol-PEG decorated gold nanorods against *S. aureus* biofilm [66]. Due to a higher hydrophobicity, cholesterol-decorated AuNRs had the highest bactericidal effect [66] (Figure 5A,B). Based on the same principle of interactions, Taheri et al. showed antibacterial effects of 1-palmitoyl-2-oleoyl-sn-glycero-3-phospho-L-serine-coated silver nanoparticles (POPS-AgNPs) against Gram-positive *S. aureus* and *S. epidermidis*, and Gram-negative *P. aeruginosa* [67]. The modifications in lipids of the biomembrane and membrane fluidization owing to the hydrophobic interactions with the nanoparticles have been confirmed in *P. aeruginosa* by chromatographic and mass spectrometric methods, using poly-unsaturated fatty acids (PUFAs) [65]. Further, Pal et al. showed that anderosin-coated AgNPs inhibited biofilms of *Klebsiella pneumoniae*, *P. aeruginosa*, *E. coli*, and *Salmonella typhi* [63]. Using NMR, fluorimetry and molecular dynamic simulations, the authors concluded that the anderosin-coated nanoparticles interacted with the bacterial membrane via electrostatic as well as hydrophobic interactions. This caused a hydrophobic phospholipid collapse in the cell membrane, leading to pore formation and bacterial cell death [63] (Figure 5A,B). In another report, Khalid et al. used rhamnolipid-coated AgNPs and proved their antimicrobial activity in early and mature biofilms of *S. aureus* and *P. aeruginosa* [68]. Rhamnolipids have a tendency to interact with the bacterial cell membrane and biofilm lipids via hydrophobic interactions. Owing to the improved hydrophobicity of rhamnolipid-coated AgNPs, and due to the amphiphilic nature of rhamnolipids, the nanoparticle-treated biofilms showed disrupted bacterial cell membranes and reduced biofilm adhesion. The authors also suggested that the amphiphilic nature of rhamnolipid causes surfactant-like action, resulting in biofilm inhibition [68]. Using a different approach, Tanvir et al. took advantage of electrostatic interactions for developing a β-galactosidase-linked AuNPs-based sensor for detection of *S. aureus*, *E. coli*, *S. enterica* and *P. aeruginosa* [69]. The phospholipids and LPS have an overall negative charge. The authors used cationic surfactant decorated gold nanoparticles (CTAB-AuNPs). Due to electrostatic attractions between CTAB and the phospholipid/LPS layers, the AuNPs were aggregated preferentially on the bacterial cell wall. In this biosensor, higher attraction and preferential aggregation on bacterial cell wall led to an increase in β-galactosidase-mediated hydrolysis of chlorophenol red-β-galactopyranoside and change in color of the solution. With such a simple technique, the authors have given indirect proof for the existence of electrostatic interactions between lipids and AuNPs [69]. Mahmoud et al. also showed higher in vitro antibacterial activity of neutral poly(ethylene glycol)-coated gold nanoparticles (PEG-AuNPs) and cationic poly(allylamine hydrochloride)-coated gold nanoparticles (PAH-AuNPs) against *S. aureus* and *P. aeruginosa*, compared to anionic poly(acrylic acid)-coated gold nanoparticles (PAA-AuNPs), owing to their electrostatic interactions with the negatively charged bacterial cell membrane [70] (Figure 5A). In a different report, the same group of authors showed an effect of hydrophilic and hydrophobic functionalized AuNPs on the biofilms of *S. aureus* and *Propionibacterium acnes* [71]. The authors suggested that the PEG moiety was responsible for improving biocompatibility and reducing non-specific protein binding in the case of neutral AuNPs (PEG-AuNPs), thereby guiding adsorption on the bacterial cell membrane. By contrast, the polystyrene moiety on hydrophobic AuNPs (PS-AuNPs) exhibited hydrophobic interactions [71]. Giri et al. synthesized AuNPs with controlled density of surface ligand to control the positive charge on the surface (cationic thioalkyl tetra(ethylene glycol)ated trimethylammonium (TTMA) and neutral tetraethylene glycol (TEGOH)-coated AuNPs) [72]. They showed that the cationic AuNPs (with 100% TTMA surface coverage) had the highest biofilm eradication activity as compared to mixed surface coverage (~20–60%TTMA and 80–40% TEGOH). The neutral AuNPs (100% TEGOH) showed the least biofilm disruption. The authors also mentioned that lesser surface coverage by TTMA (~20%) is sufficient to cause toxicity to *S. aureus* and *P. aeruginosa* biofilms. The authors proposed that, along with electrostatic interactions, the hydrophobicity of TTMA-AuNPs plays a role in biofilm inhibitory activity [72]. Similarly, in a report published by Morales-Avila et al., AuNPs and AgNPs conjugated to ubiquicidin peptide showed electrostatic interaction with lipoteichoic acid, phospholipids and LPS, while showing synergistic antimicrobial action against *E. coli* and *P. aeruginosa* [60] (Figure 5A). Overall, the biofilm lipids interact with metallic nanoparticles mainly by electrostatic and hydrophobic interactions.

### 3.5. Effect of Physicochemical Properties of Nanoparticles and Biofilms on Interactions

As stated before, when AuNPs and AgNPs come in contact with any biological media, the components of that media (e.g., proteins, peptides, amino acids) have a tendency to get adsorbed on the surface of nanoparticles. This layer is termed as the “nanoparticle corona” [21]. In many cases, AuNPs and AgNPs are artificially decorated with functional moieties to enhance a specific activity, e.g., antibacterial action. Hence, when estimating overall antimicrobial activity, most commonly, one has to take into account the effects of the nanoparticle itself and its corona [21]. The biofilm itself, as a target for antimicrobial NPs, also varies dramatically from species to species, and its composition can be further modified depending on the environment [1,3,5]. In this section we will review the physicochemical properties of both the nanoparticles and the biofilm component and provide an overview of their impact on NP–biofilm interactions [21].

Nallathambi et al. have shown the effects of size and concentration of AgNPs on their antibacterial activity against *P. aeruginosa* [73]. Comparing AgNPs of average diameter of 13 and 90 nm, the authors showed that the smaller nanoparticles, 13 nm, achieved higher intracellular concentration in *P. aeruginosa*, due to easier passive diffusion. In addition, nanoparticle internalization increased linearly with concentration, suggesting a direct impact of the dose or concentration of nanoparticles fed to the bacterial culture. Further, the authors also reported a nanoparticle size dependent efflux activity of MexAB-OprM efflux transporter of *P. aeruginosa*, whereby the transporter removed larger nanoparticles more effectively [73]. In an another report, Peulen and Wilkinson showed faster diffusion of smaller nanoparticles (<50 nm) through the pores of biofilms [74]. Apart from size and concentration, the shape of nanoparticles also matters for their antibacterial activity. In separate reports, Penders et al. and Cheon et al. proved the dependency of antimicrobial activity on the shape of AuNPs and AgNPs respectively [75,76]. Penders et al. prepared gold nanospheres (AuNS), gold nanostars (AuNSts) and gold nanoflowers (AuNFs) and checked their activity against *S. aureus* biofilm. Among these, AuNSts and AuNFs exhibited the highest antimicrobial activity. The authors attributed this to increased surface area because of different shapes, which ultimately provides a higher probability of interactions with the bacterial cell membrane, as well as with other biofilm components. The most active AuNFs had protrusions on the surface, which according to the authors contributed significantly to the interaction with bacterial membranes [75]. In the case of AgNPs, Cheon et al. prepared silver nanospheres (AgNS), silver nanodiscs (AgNDs) and silver nanotriangle plates (AgNtr). All of these nanoparticles showed significant antimicrobial activity against *E. coli*, but not against *P. aeruginosa* and *S. aureus*. Against *E. coli*, among these nanoparticles, AgNS showed the highest activity. The authors speculated that AgNS releases higher amounts of silver ions, which interact with planktonic bacteria and kill them. Thus, as compared to AgNDs and AgNTr, which had lower surface areas and thus lower rates of silver ion release, AgNS had the highest activity [76]. In all aforementioned reports, the authors also showed a concentration-dependent rise in nanoparticle activity against bacteria [73,74,75,76]. To show the impact of surface charge of nanoparticles on antibiofilm activity, Badawy et al. synthesized uncoated AgNPs, neutral AgNPs, cationic AgNPs and anionic AgNPs [54]. Among these, anionic AgNPs showed the weakest antibacterial activity against Gram-positive *Bacillus* species, presumably due to electrostatic repulsion. The activity was higher in the case of neutral and uncoated AgNPs, due to weaker electrostatic repulsion. Cationic AgNPs exhibited the strongest antibacterial activity [54]. Similarly, in separate reports, Giri et al. and Pajerski et al. showed that the surface charge of AuNPs had an impact on their biofilm inhibition activity [49,72]. These findings strongly suggest that the surface chemistry and surface charge control the nature of nanoparticle–biofilm interactions and thereby modulate their antimicrobial activity.

In the case of biofilm properties, the most important factors determining the interaction with nanoparticles is biofilm maturity and thickness. As shown by Peulen and Wilkinson, mature biofilms have denser EPS [74]. Thus, the number of pores and the pore sizes are dramatically reduced in denser, mature biofilms, making it difficult for nanoparticles to penetrate the biofilm. Accordingly, the antimicrobial activity of AuNPs and AgNPs is higher in younger biofilms. This effect is related to better adhesion to the biofilm surface, enhanced penetration and stronger interactions with all biofilm components in the interior. Mitzel and Tufenkji reported that the surface of mature biofilms shows an electrostatic repulsion of sterically stabilized silver nanoparticles (PVP-AgNPs) [56]. Radzig et al. reported a similar observation for AuNPs and gold ions, when used against mature biofilms of *E. coli* AB1157 [27]. Mitzel et al. reported that the hydrophobicity of *P. aeruginosa* 9027^TM^ biofilm and *P. aeruginosa* PAO1 biofilm had an impact on their interaction with nanoparticles [77]. They reported that the transport, deposition and retention kinetics of the surface-modified polystyrene nanoparticles changed depending on the relative hydrophobicity of the two biofilms. Although their report was published for polystyrene nanoparticles [77], the impact of hydrophobicity of biofilms is relatable to AuNPs and AgNPs. For example, lipid-coated AuNPs and AgNPs are known to modify the hydrophobicity of biofilms [66,67,68]. Bacterial biofilms are capable of maintaining a specific pH value in their matrix. The pH of the biofilm microenvironment also governs its interactions with nanoparticles. For example, Hu et al. and Wu et al. have shown improved interactions and penetration of AuNPs and AgNPs in methicillin-resistant *S. aureus* biofilm [78,79]. Hu et al. used 11-mercaptodecanoic acid and (10-mercaptodecyl)-trimethylammonium bromide coated AuNPs and showed that due to the zwitterionic nature of ligands on the surface of nanoparticles, Van der Waals attraction, hydrogen-bonding, hydration repulsion and electrostatic repulsion remained balanced, keeping nanoparticles stably dispersed at neutral pH. However, in the acidic microenvironment of a biofilm, the ligands on AuNPs underwent charge reversal, leading to improved electrostatic attraction and aggregation of nanoparticles. The authors used near infrared radiation (NIR) along with aggregates of gold nanoparticles (photothermal method) and showed eradication of the biofilm [78]. Similarly, Wu et al. used poly(ethyleneglycol)-poly(aminopropyl imidazole-aspartate)-polyalanine-coated silver nanoassemblies. In their report, they proved that at neutral pH, the ligand-coated nanoassemblies were stable and uniformly dispersed. However, upon entering the acidic microenvironment of the biofilm, the ligands underwent charge reversal, leading to improved electrostatic repulsion and disassembly of silver nanoclusters and reduction in nanoparticle size (from ~150 nm to ~8 nm). This reduction greatly accelerated the dissolution of nanoparticles and silver ion leaching, which eventually eradicated the methicillin-resistant *S. aureus* biofilm [79]. These reports prove that biofilm pH has a significant impact on its interaction with metallic nanoparticles [78,79]. Biofilm surface chemistry is another factor that could alter the course of biofilm–nanoparticle interactions. Gram-negative and Gram-positive bacteria have significant differences in their cell wall structure. Gram-positive bacteria have a thick peptidoglycan layer covered with wall teichoic acid and lipoteichoic acid layers facing the exterior. By contrast, Gram-negative bacteria have an outer cell membrane covered with lipopolysaccharides facing the exterior (outside of a thin peptidoglycan layer). This structural difference leads to very different surface chemistries of biofilms formed by these two classes of bacteria and impacts their interactions with AuNPs and AgNPs [49,53]. In addition, the ionization of carboxylate and phosphate groups in teichoic acid and lipoteichoic acid of Gram-negative bacterial cell walls as well as in lipopolysaccharides of Gram-positive bacterial cell walls depends on the pH of the biofilm microenvironment. This factor could change the nature of interactions between nanoparticles and the bacterial cell wall. Overall, the physicochemical properties of both the nanoparticles and biofilm significantly participate in the nano-bio interactions, antimicrobial action and resistance.

## 4. Interactions Involved in Biofilm Resistance to the Nanoparticles

As stated in previous paragraphs, AuNPs and AgNPs show antimicrobial activity against a broad spectrum of bacteria via multiple mechanisms. Therefore, these nanoparticles have turned out to be an excellent choice over conventional antibiotics, against which many bacterial strains have developed resistance. Initially it was thought that, due to the diversity in the mechanism of antimicrobial activity of AuNPs and AgNPs, the probability of bacteria developing resistance against these nanoparticles was very low. However, recent research suggests otherwise [80]. Biofilm-forming bacteria show resistance to these nanoparticles by genetic mutations (e.g., up-regulation of efflux pump proteins), molecular interactions (e.g., electrostatic interactions) or by biofilm adaptation (e.g., increased secretion of EPS or development of resistance after many culture steps) [80].

Faghihzadeh et al. showed that acute and chronic exposure to AgNPs causes genetic and phenotypic changes in *E. coli,* which ultimately leads to resistance [81]. One of the changes observed by the authors was a modification of composition and amount of EPS secreted by *E. coli*. Specifically, through up-regulation of capsular polysaccharide regulon gene (*cpsB* gene), the amount of biofilm polysaccharides was increased after exposure to AgNPs [81]. Additionally, the ATP-dependent efflux of silver was also increased due to overexpression of copper transporter gene (*copA* gene). Randall et al. reported two mechanisms for resistance to antimicrobial action of AgNPs and silver ions [82]. In their work, the authors showed that *E. coli* negates the action of AgNPs through expression of a silver sequestering protein (SilE) and increased efflux via SilCFBA efflux transporter [82]. Interestingly, Panáček et al. showed that *E. coli* and *P. aeruginosa* resistance to AgNPs occurs only via phenotypic changes and does not involve genetic changes [83]. In two different strains of *E. coli* (CCM3954 and 013) and in *P. aeruginosa*, the authors showed development of resistance to AgNPs after a number of culture steps. For *E. coli* and *P. aeruginosa*, after 13 culture steps, the authors observed 16-fold rise and 32-fold rise in minimum inhibitory concentration values, respectively. The resistance developed specifically against AgNPs and not against ionic silver. The authors reported that the *E. coli* secreted excess of flagellin, which induced AgNP aggregation and sedimentation. The authors attributed Van der Waals attraction and electrostatic repulsion to this aggregation phenomenon, based on the DLVO theory [83]. With the help of elemental mapping, the authors suggested that Van der Waals attraction forces dominate over repulsion forces in the case of flagellin-coated silver nanoparticles, causing their aggregation and sedimentation [83]. In addition to these reports, Siemer et al. revealed that the corona on the surface of AgNPs affects the interactions between the nanoparticles and the bacterial cells along with other parameters such as the surface chemistry of AgNPs and the bacterial cell wall composition [84]. The authors suggested that, via electrostatic repulsive interactions, the corona reduces the activity of AgNPs [80,84]. Depending on the surrounding environment, the ionic composition of the biofilm microenvironment changes. Additionally, the natural organic matter accompanying the biofilms contains several cations and anions, which have shown to significantly impact the interaction of nanoparticles with biofilm components and bacterial cell. For example, the electrostatic interaction between negatively charged ions, such as chloride ions, hampered the antimicrobial action of AgNPs and silver ions released from those nanoparticles [80,85,86]. Due to increased dissolution of AgNPs, the activity initially increased, but over a long period of time, due to the binding of silver ions to chloride ions, the activity eventually reduced [85,86]. Further, in two separate reports, Zhang and Oyanedel-Craver as well as Jin et al. showed that divalent cations (Ca^2+^ and Mg^2+^) formed ion bridges between AgNPs and promoted Van der Waals attraction, causing NP aggregation and sedimentation in an aqueous environment. This reduced the antimicrobial activity of those nanoparticles against *E. coli*, *B. subtilis*, and *Pseudomonas putida* biofilms significantly [87,88]. The participation of biofilm components in sequestration of AuNPs and AgNPs has also been revealed [80,89,90]. In this sequestration, electrostatic, hydrophobic and hydrogen-bonding interactions are involved [80,89,90]. Surprisingly, there are very limited published data regarding biofilm resistance to AuNPs. To note among such few reports, Elbehiry et al. showed resistance of *S. aureus* strains to 10 and 20 nm AuNPs and AgNPs [91]. However, the exact mechanism of resistance was not elucidated by the authors. Further, it has been suggested that the reducing components from biofilm and planktonic bacteria such as acids, sugars, alcohols, as well as the protein components of biofilm tend to stabilize the gold and silver ions by masking their surface [80]. In addition, small organic components such as phenazine compounds (e.g., pyocyanin) from *P. aeruginosa* sequester ions released from nanoparticles to protect the bacterial cells [80]. Some bacteria, e.g., *Enterobacter cloacae*, can convert gold ions into metallic nanoclusters (Au^3+^ to Au-nanocluster), rendering them ineffective against biofilms [80,92,93]. This phenomenon of conversion of ions into nanoclusters has yielded a robust, cheap and environmentally friendly process for synthesizing AuNPs and AgNPs using bacterial cells. Other than these mechanisms, the biofilms can also achieve resistance to AuNPs and AgNPs by constructing a physical barrier that prevents all molecular interactions [80]. The protein (e.g., flagellin from *P. aeruginosa*) and polysaccharide (e.g., alginate or Pel from *P. aeruginosa*) components of the biofilm form a dense network with very low pore sizes, which serves as a strong physical barrier against entry of nanoparticles with sizes >10 to 20 nm. Such a barrier prevents passive diffusion of nanoparticles and reduces their antimicrobial action [57,83,93]. In general, the use of AuNPs and AgNPs is also hampered by bacterial resistance, and in that sense they are similar to classical antibiotics. However, the resistance mechanisms to nanoparticles are different, and in some case non-genetic, which probably means they will be easier to circumvent.

## 5. Conclusions and Perspective

Emergence of multiresistant bacterial strains has created an alarming situation that has driven the research in the direction of finding novel therapies to combat the infections and diseases associated with bacterial biofilms. Among the new modes of therapies, metallic nanoparticles, particularly gold and silver nanoparticles, have gained popularity because of their unique advantages such as small size, high surface area to volume ratio, biocompatibility and amenability to surface modifications. Deciphering the mechanistic details behind their antimicrobial actions has led to an increased understanding of the involvement of biofilm components in interaction with these nanoparticles and their respective metal ions. The biofilm components (nucleic acids, proteins, polysaccharides and lipids) interact with AuNPs and AgNPs via electrostatic interactions, hydrophobic interactions, hydrogen-bonding interactions, Van der Waals interactions, ionic interactions and π–π interactions. The nature of these interactions depends on the physicochemical properties of the biofilm as well as the nanoparticles. Modifications in the properties of the nanoparticles by biofilm components and the biofilm microenvironment also change the nature of these interactions, which in turn affect their antimicrobial activity. Our literature survey suggests that the probability of occurrence of nanoparticle–biofilm interactions roughly varies in decreasing order of electrostatic interactions > hydrophobic interactions > Van der Waals interactions ~ ionic interactions ~ hydrogen-bonding interactions > π–π interactions. Electrostatic interactions dominate all other interactions in terms of their contribution to the antimicrobial activity of nanoparticles as well as to the biofilm resistance. The main approach to modulating the nature of nanoparticle–biofilm interactions is making AuNPs and AgNPs highly selective against a single component of the biofilm via bioconjugation techniques. AuNPs and AgNPs decorated with various chemical moieties have proven to fair better than uncoated nanoparticles in terms of their interactions with the biofilm and antibacterial activity.

There are plenty of reports of successful attempts of biofilm treatment by AuNPs and AgNPs at preclinical phases. Despite their success in preclinical testing, the antimicrobial nanotherapeutic approaches still face a great hurdle of clinical testing [94]. The important caveat in finding new antimicrobial compounds is biosafety and biocompatibility. Any novel therapeutic molecule or device should be biosafe and biocompatible to the host while eradicating biofilm successfully. In spite of many reports proving in vitro and in vivo safety of AuNPs and AgNPs under various experimental conditions, these nanoparticles still pose as threat to our ecosystem [95]. AuNPs and AgNPs remain in air, water and soil for longer durations and may exert toxicological effects on humans and other species of animals and plants [95]. Thus, apart from clinical testing, the risk assessment and risk management with respect to environmental safety of these nanoparticles stand as strong barriers for their real-life applications [95]. Several regulatory agencies, for example the Food and Drug Administration (USA), European Medicines Agency (Europe), and Pharmaceuticals and Medical Devices Agency (Japan), have established guidelines for manufacturing and characterizing nanoparticles as well as offer risk assessment, nano-waste management and nano-waste disposal guidance [96,97,98,99]. Such stringent control on nanoparticle-related research from regulatory authorities ensures their development and progress towards effective therapeutic modality. Close association of research groups with regulatory authorities and regulatory counselors at each step of research may prove fruitful in generating patient-compliant as well as regulatory-compliant nanomedicine. Other impeding issues involve industrial scale-up of nanoparticle synthesis and development of sensitive assays to detect the smallest quantity of nanoparticles in environmental samples, which can be solved by critical control on manufacturing process.

In addition to these regulatory and biosafety-related issues, AuNPs and AgNPs also face some experimental challenges at the laboratory level. The possibility of biofilm resistance to this novel therapeutic approach is a considerable challenge in the application of nanoparticles in antimicrobial therapy. Many experimental treatment modalities that were initially effective have shown a drop in antimicrobial effect due to development of bacterial resistance. The mechanisms of resistance to nanoparticles are still poorly understood. However, the use of combination therapy (e.g., cocktail of metallic nanoparticles with classical antibiotics or nanoparticles conjugated with additional antimicrobial molecules (e.g., AMPs)) has significantly reduced this problem. Given the complexity of biofilms, the use of multi-targeted approaches seems to be a sound strategy going forward. Combinatorial strategies entail reducing the dose of administered nanoparticles and antibiotics as well as a benefit from synergistic effects. Another drawback of the available literature is the experimental setup commonly used in the reported studies, which typically involve single-species biofilms. In reality, naturally occurring biofilms consist of several bacterial species that maintain harmony within the biofilm and survive together. The composition of mixed biofilm differs significantly as compared to the biofilm of a single bacterial species. This could change the nature of interactions between nanoparticles and biofilm components. Modifying experimental designs from examining single-species biofilms to working with multiple-species biofilms will be helpful in deducing the exact interactions between nanoparticles and the components of native, medically relevant biofilms. Notwithstanding these few drawbacks, technological development in the area of nanotherapeutics over the last two decades underpins a tremendous potential of gold and silver nanoparticles for developing new antimicrobial applications.

## Figures and Tables

**Table 1:** Summary of interactions between biofilm components and gold/silver nanoparticles.

**Interactions between Nanoparticles and Biofilm Components during Antimicrobial Action of AuNPs and AgNPs**	
**Component**	**Type of Interaction**
Nucleic acids	Electrostatic interactions, Van der Waals interactions, Hydrophobic interactions, Gold–Sulphur (Au-S) chemistry
Proteins	Electrostatic interactions, Hydrophobic interactions, Hydrogen-bonding interactions, Van der Waals interactions, π–π interactions, Gold–Sulphur (Au-S) chemistry
Polysaccharides	Electrostatic interactions, Hydrophobic interactions, Hydrogen-bonding interactions, Electrosteric repulsion interactions
Lipids	Hydrophobic interactions, Electrostatic interactions
**Interactions between Nanoparticles and Biofilm/Planktonic Bacterial Cells during Resistance to the Antimicrobial Action of AuNPs and AgNPs**	
**Component**	**Type of Interaction**
Proteins	Electrostatic interactions, Van der Waals interactions, Hydrophobic interactions, Hydrogen-bonding interactions
Polysaccharides	Electrostatic interactions, Physical barrier for nanoparticles (no interactions involved)
Cations	Ionic interactions
Anions	Electrostatic interactions, ionic interactions

## Abbreviations

3D	Three-dimensional
Ag^+^	Silver ions
AgNDs	Silver nanodiscs
AgNPs	Silver nanoparticles
AgNS	Silver nanospheres
AgNtr	Silver nanotriangular plates
AIP-I	Autoinducing peptide-I
AMPs	Antimicrobial peptides
ATP	Adenosine triphosphate
Au^+^	Gold ions
Au-Ag-NPs	Bimetallic hybrid gold–silver nanoparticles
AuNFs	Gold nanoflowers
AuNPs	Gold nanoparticles
AuNRs	Gold nanorods
AuNS	Gold nanospheres
AuNSts	Gold nanostars
BAP	Biofilm-associated protein
bPEI	Branched polyethylenimine
bPEI-AgNPs	Branched polyethylenimine coated silver nanoparticles
bPEI-AuNPs	Branched polyethylenimine coated gold nanoparticles
BslA	Biofilm surface layer protein A
Chitosan-AgNPs	Chitosan-coated silver nanoparticles
*copA*	Copper transporter gene
*cpsB*	Capsular polysaccharide regulon gene
CTAB	Cetyl trimethylammonium bromide
CTAB-AuNPs	Cetyl trimethylammonium bromide coated gold nanoparticles
Cyclic-di-GMP	Bis-(3′,5′)-cyclic dimeric guanosine monophosphate
DLS	Dynamic light scattering
DLVO theory	Derjaguin–Landau–Verwey–Overbeek theory
DSPE	1,2-Distearoyl-sn-glycero-3-phosphorylethanolamine
DSPE-AuNPs	1,2-Distearoyl-sn-glycero-3-phosphorylethanolamine coated gold nanoparticles
EbpA	*Enterococcus faecalis* pilus tip
eDNA	Extracellular deoxyribonucleic acid
EDTA	Ethylenediaminetetraacetic acid
EPS	Extracellular polymeric substances
FapC	Amyloid-like fimbriae protein from *Pseudomonas* species
FnBPs	Fibronectin binding proteins
HSP-18	Heat shock protein-18
LasI	Acyl-homoserine-lactone (AHL) synthase
LasR	Transcriptional activator protein required for activation of elastase structural gene (LasB)
LPS	Lipopolysaccharides
LpxC	UDP-3-*O*-(R-3-hydroxymyristoyl)-*N*-acetylglucosamine deacetylase (encoded by lpxC gene)
MexAb-OprM	Outer membrane efflux protein from *P. aeruginosa*
MreB	Cell shape determining protein of *E. coli*
MvfR	Multiple virulence factor regulator protein from *P. aeruginosa*
NIR	Near infrared
NMR	Nuclear magnetic resonance
NRs	Nanorods
PAA	Poly(acrylic acid)
PAA-AuNPs	Poly(acrylic acid)-coated gold nanoparticles
PAH-AuNPs	Poly(allylamine hydrochloride)-coated gold nanoparticles
PEG	Poly(ethyleneglycol)
PEG-AuNPs	Poly(ethyleneglycol)-coated gold nanoparticles
PEG-PSB-PALA-AgNAs	Poly(ethyleneglycol)-poly(aminopropyl imidazole aspartate)-polyalanine-coated silver nanoassemblies
POPS-AgNPs	1-palmitoyl-2-oleoyl-sn-glycero-3-phospho-L-serine-coated silver nanoparticles
PS-AuNPs	Polystyrene-coated gold nanoparticles
PT	Phosphorothioation
PUFAs	Polyunsaturated fatty acids
PVP	Polyvinylpyrrolidone
QS	Quorum sensing
QscR	Quorum sensing control repressor protein
RhlR	Regulatory protein required for transcriptional activation of gene associated with rhamnosyltransferase
RIP	RNA-III inhibiting peptide
ROS	Reactive oxygen species
silCFBA	Active efflux transporter complex of proteins (ATPase and silver binding protein SilE) responsible for efflux of silver ions
silE	Silver binding/sequestering protein
TEGOH	Tetraethylene glycol
TTMA	Thioalkyl tetra(ethylene glycol)ated trimethylammonium
Vfr	cAMP-activated global transcriptional regulator which controls virulence factor gene expression
